# Impact of The Endometrioma on Ovarian Response and Pregnancy Rate in *In Vitro* Fertilization Cycles

**Published:** 2014-03-09

**Authors:** Mahnaz Ashrafi, Taravat Fakheri, Kiandokht Kiani, Maria Sadeghi, Mohammad Reza Akhoond

**Affiliations:** 1 Department of Endocrinology and Female Infertility at Reproductive Biomedicine Research Center, Royan Institute for Reproductive Biomedicine, ACECR, Tehran, Iran; 2 Department of Obstetrics and Gynecology, Iran University of Medical Sciences, Tehran, Iran; 3 Department of Obstetrics and Gynecology, Kermanshah University of Medical Sciences, Kermanshah, Iran; 4 Vali-e-Asr Reproductive Health Research Center, Tehran University of Medical Sciences, Tehran, Iran; 5 Statistics Department, Mathematical Science and Computer Faculty, Shahid Chamran University, Ahwaz, Iran

**Keywords:** Endometrioma, Ovulation Induction, In Vitro Fertilization, Pregnancy Rate

## Abstract

**Background:**

Our objective was to evaluate the effect of ovarian endometrioma on ovarian stimulation outcomes in *in vitro* fertilization cycles (IVF).

**Materials and Methods:**

In this prospective cohort study, we followed 103 patients who underwent intra-cytoplasmic sperm injection (ICSI) procedures over a 24-months period. The study group consisted of 47 infertile women with either unilateral or bilateral ovarian endometrial cysts of less than 3 cm. The control group consisting of 57 patients with mild male factor infertility was candidate for ICSI treatment during the same time period as the study groups. Both groups were compared for number of oocytes retrieved, grades of oocytes, as well as embryo quantity and quality.

**Results:**

Our results showed similar follicle numbers, good embryo grades (A or B) and pregnancy rates in the compared groups. However, patients with endometrioma had higher gonadotropin consumption than the control group. The mean number of retrieved oocytes in patients with endometrioma was significantly lower than control group (6.6 ± 3.74 vs. 10.4 ± 5.25) (p<0.001). In addition, patients with endometrioma had significantly lower numbers of metaphase II (MII) oocytes (5 ± 3.21) than controls (8.2 ± 5.4) (p<0.001). In patients with unilateral endometrioma, there were no significant differences in main outcome measures between normal and involved ovaries in the patients with endometrioma.

**Conclusion:**

Patients with ovarian endometrioma had poor outcome. They showed poor ovarian response with lower total numbers of retrieved oocytes and lower MII oocytes during the stimulation phase; however, it does not affect the total number of embryos transferred per patient, quality of embryos, and pregnancy rate per patient.

## Introduction

At present, approximately 20-35% of all patients undergoing *in vitro* fertilization (IVF) are diagnosed with endometriosis (1, 2) among whom about 30-40% suffer from ovarian endometrioma (3, 4). The impact of ovarian endometrioma on assisted reproductive technologies (ART) results is a controversial issue. A previous meta-analysis (5) has shown reduced pregnancy rates in women with ovarian endometrioma who underwent IVF treatments when compared to patients with other infertility causes. However, other studies have not confirmed this finding (6-8). In addition, some studies have shown that ovarian endometrioma could adversely affect the number of oocytes retrieved (7, 9), oocyte quality, fertilization rate (5, 10), embryo quality and implantation rate (9, 11, 12). Kumbak et al. (13) have also reported poor embryo quality in patients with endometriosis; yet, there have been no effect on pregnancy rate. Other studies also found no adverse effect of ovarian endometrioma on pregnancy success rates (14-16).

Due to these conflicting results, the optimum management of ovarian endometrioma in IVF/ intra cytoplasmic sperm injection (ICSI) cycles is not clear (14, 17). Some authors believe that ovarian endometriomas should be removed before the IVF cycle (18); however, others have shown that excision of an ovarian endometrioma before an IVF cycle is likely to lead poor responses to ovarian stimulation and to impact fertility outcomes (19, 20). 

The purpose of the present study was to evaluate the effect of ovarian endometriomas on ovarian response and IVF/ ICSI outcomes when compared to patients with mild male factor infertility.

## Materials and Methods

This cohort study was performed at Royan Institute for Reproductive Biomedicine (Tehran, Iran) over a 24-months period between March 2005 and December 2007. We recruited a total of 104 women who were candidates for IVF/ICSI and fresh embryo transfers.

The study group consisted of 47 infertile women with either unilateral or bilateral ovarian endometrial cysts of less than 3 cm, based upon transvaginal sonographic diagnosis with diffuse low-level echoes without neoplastic or acute hemorrhage features. Patients had no histories of any ovarian surgeries. All ultrasound tests were performed by two expert technicians using an EUB 6000 (HI- TACHI, Japan) equipped with a 6 MHZ curvilinear color doppler probe. Technicians performed trans-vaginal ultrasounds between days 1 and 8 of the cycle, before starting ovarian stimulation. The location and dimension of the endometriomas were recorded at this time.

Patients with endometriomas larger than 3 cm underwent endometriotic cystectomy, via laparoscopic surgery. These patients were not included at the study. There was no patient with cystectomies, defined as a laparoscopic surgery applied for women with small ovarian endometriomas.

The control group consisting of 57 patients with mild male factor infertility was candidate for ICSI treatment during the same time period as the study groups. Mild male factor infertility was defined as the presence of at least 1 million motile sperm after processing.

All patients in both groups had indications for IVF/ICSI treatments, without any previous attempts.

Exclusion criteria for both groups were as follows: i. previous history of any systematic disease or malignancy, ii. basal follicle-stimulating hormone (FSH) more than 15 mIU/ml, iii. history of three or more unsuccessful IVF attempts, and iv. ovarian endometrioma less than 3 cm.

The study was approved by the Ethics Committee of Royan Institute for Reproductive Biomedicine. A written informed consent was obtained from all individuals before participation.

Stimulation protocol for all patients was according to the standard long protocol. All patients underwent oral contraceptive suppression starting from the 2^nd^ or 3^rd^ day of the menstrual cycle. Gonadotropin-releasing hormone agonist (GnRH agonist) suppression with Busereline (500 µg, Suprefact; Aventis Pharma Deutshlan, Frankfurt, Germany) was performed via subcutaneous injection starting on the 21^st^ day of the menstrual cycle. We began gonadotropin stimulation 14 days after subcutaneous GnRH agonist injection with daily dose of 150 IU of recombinant FSH (Gonal F; Serono, Geneva, Switzerland). The dose and duration of FSH treatment were adjusted by monitoring follicular development using ultrasound and estradiol levels. In both groups, gonadotropin stimulation continued until 2-3 follicles with a mean diameter of ≥17 mm were achieved. Then, 10000 IU of human chorionic gonadotropin (hCG; Choriomon; IBSA, Lugano, Switzerland) was administered, while oocyte retrieval was performed 34-36 hours later by a skilled gynecologist. Only normal follicles were punctured at the time of oocyte retrieval. During the procedure, every effort was made to avoid puncturing the endometrioma. If endometriomas were accidentally punctured, the procedure was interrupted and resumed after the needled was changed.

Metaphase II (MII) oocytes were injected using ICSI procedure. Normal fertilization was confirmed when two distinct pronuclei were present within 16-18 hours following oocyte injection.

### ICSI procedure

In our ICSI cycles, cumulus-enclosed oocytes were treated with 0.1% hyaluronidase, and the cumulus cells were mechanically removed by pipetting. Oocyte maturation stage and morphology were assessed under an inverted microscope (Olympus, Tokyo, Japan) at ×400 magnification. Oocytes were classified as MII oocyte (mature oocyte), metaphase I oocyte, or prophase I oocyte. Good quality cleaved embryos (21) were transferred 2-3 days after oocyte retrieval.

Progesterone supplementation (400 mg twice a day; Aburaihan Co., Tehran, Iran) was provided at time of oocyte retrieval until the day of β-hCG assay. After obtaining a positive pregnancy test result, it was continued until the 10^th^ week of gesta- tion.

The initial dose, total dose and days of gonadotropin, endometrium thickness, and concentration of estradiol (E_2_) on the days of hCG injection were recorded. Numbers of retrieved eggs, rate of cleavage, numbers of embryos grades A and B obtained, scores of the transferred embryos, as well as rates of clinical pregnancy and implantation were calculated.

### Outcome

Primary endpoints were ovarian response and oocyte quality and quantity. The quality of embryos, biochemical, clinical pregnancy and implantation rates were other outcomes of interest. We defined the fertilization rate as the ratio of the number of embryos formed relative to the number of MII oocytes injected. The maturation rate was the ratio of MII oocytes to the number of total retrieved oocytes. Clinical pregnancy was a positive pregnancy test result followed by the presence of a gestational sac on trans-vaginal ultrasound, 4 weeks after transfer. The pregnancy rate in the present study was calculated by dividing the number of clinical pregnancies detected by the number of patients (clinical pregnancy per patient). The implantation rate was the number of gestational sacs visualized by trans-vaginal pelvic ultrasound per embryos transferred. In addition, we compared the normal and involved ovaries in patients with unilateral endometrioma.

### Statistical analysis

Analysis was performed using the SPSS (version 13.0; SPSS Inc., Chicago, IL, USA) statistical software. Between-group differences of normally distributed continuous variables were assessed by student’s t test, whereas the Mann-Whitney U test was used for the abnormal distributed data. Significant differences were evaluated by the chisquare test to compare non-continuous variables. In inter-group comparison, the paired t test analysis and Wilcoxon Signed Ranks test were applied for patients with unilateral endometrioma and for nonparametric cases, respectively. Data were expressed as mean ± standard deviation (SD). Statistical significance was considered when p<0.05. 

## Results

The baseline characteristics including age, duration of infertility, and basic concentrations of E_2_ and FSH level were similar between two groups (Table 1).

The mean number of retrieved oocytes in patients with endometrioma and in control group were 6.6 ± 3.74 vs. 10.4 ± 5.25, respectively (p<0.001). As seen in table 2, the numbers of MII oocytes were significantly lower in patients with endometrioma (5 ± 3.21) as compared to the control group (8.2 ± 5.4).

The thickness of the endometrium, follicle numbers and good quality embryos (grades A or B) were also comparable between the two groups (p>0.05). Both groups had similar implantation and pregnancy rates (Table 2). However, patients with endometrioma had higher gonadotropin consumption as compared with the control group. We had no severe ovarian hyperstimulation syndrome (OHSS) in both groups.

In patients with unilateral endometrioma, we compared outcomes between the affected ovary and healthy contra lateral ovary. There were no significant differences in terms of main outcome measures between the normal and involved ovaries (Table 3).

**Table 1:** Comparison of the baseline characteristics between patients with endometrioma and control groups


	Patients with endometrioma(n= 47)	Control group(n= 57)	P value(%95 CI)

**Age (Y)**	31.9 ± 4.01	30.6 ± 4.71	0.124
**Duration of infertility(Y)**	8.3 ± 5.06	7.2 ± 5.18	0.281
**Basal FSH level (IU/Lit)**	6.7 ± 2.76	6.2 ± 2.10	0.362
**Basal LH level (IU/Lit)**	6.5 ± 3.87	5.5 ± 3.27	0.159
Basal estradiol level (Pg/ml)^*^	45.0(2.9-1100.0)	38 (1.4- 4800)	0.906


Data are expressed as mean ± SD, otherwise it is reported. *; Median(Min-Max) and Mann-Whitney test is used.

**Table 2:** Comparison of ICSI cycles outcomes between the patients with endometrioma and control groups


	Patients with endometrioma(n= 47)	Control group(n= 57)	P value

**Total number of gonadotropin ampoules**	31.8 ± 13.03	24 ± 9.35	0.001
**Endometrium thickness (mm)**	9.3 ± 2.06	9.4 ± 1.8	0.802
**Follicle number**	13.4 ± 11.4	12.7± 10.6	0.736
**Total number of oocytes retrieved**	6.6 ± 3.7	10.4 ± 5.2	<0.001
**MII oocytes retrieved**	5.04 ± 3.2	8.4 ± 5.1	<0.001
**Total number of embryos transferred**	3.2 ± 0.9	2.7 ± 0.9	0.040
**Total formed embryos **	3.8 ± 2.5	4.9 ± 2.3	0.084
**Good quality formed embryos**	2.9 ± 2.3	2.6 ± 3.1	0.588
**Fertilization rate (%)**	173/237 (73.0)	283/480 (59.0)	<0.001
	OR=1.882 (1.340-2.643)*		
**Maturation rate (%)**	237/311 (76.2)	480/594 (80.8)	0.105
	OR=0.761 (0.546-1.060)*		
**Cancellation rate (%)**	4 (8.5)	2(3.5)	0.276
	OR=2.558 (0.447-14.628)*		
**Implantation rate (%)**	20/133 (15.0)	17/143 (11.9)	0.443
	OR=1.312 (0.655-2.628)*		
**Clinical pregnancy rate (%)**	15 (37.5)	14 (26.4)	0.253
	OR=0.6729 (0.284-1.59)*		
**Multiple pregnancy rate (%)**	4 (28.6)	3 (18.8)	0.526
	OR=1.73 (0.31, 9.57)*		


Data are expressed as mean ± SD. OR; Odds Ratio and *; 95% Confidence Interval for odds ratio.

**Table 3:** Comparison of the clinical outcomes between the normal and involved ovaries in the patients with unilateral endometrioma


	Ovary with endometrioma(n= 37)	Normal ovary(n= 37)	P value (% 95 CI)

**Follicle number **	7.02 ± 6.9	6.6 ± 5.8	0.532 (-0.957,1.822)
**Total number of oocytes retrieved **	2.8 ± 2.4	3.4 ± 2.7	0.368(-1.744,0.663)
**MII oocytes retrieved**	2.05 ± 2.13	2.3 ± 2.24	0.572(-1.354,0.759589)
**Maturation rate**	57/76(75.0%)	76/87(87.4%)	0.042
	OR=0.434 (0.192-0.984)*		
**Fertilization rate**	76/105(72.4%)	87/125(69.6%)	0.644
	OR=1.145 (0.645-2.030)*		
**Total formed embryos **	1.5 ± 1.54	2.05 ± 1.84	0.226 (-1.358,0.331)
**Total formed embryos grade A **	0.5 ± 0.73	0.8 ± 1.2	0.162 (-0.719,0.125)
**Total formed embryos grade B **	0.5 ± 0.76	0.8 ± 0.8	0.094 (-0.64,0.053)
**Total formed embryos grade C **	0.4 ± 0.86	0.5 ± 1.16	0.833 (-0.569,0.461)


Data are expressed as mean ± SD, otherwise it is mentioned.

## Discussion

In the present study, the number of retrieved oocytes and MII oocytes were significantly lower in endometrioma patients than the controls; however, the number of good quality embryos (per MII injected oocyte) was comparable in both groups.

These results were consistent with previous investigations that found a lower ovarian response to gonadotropin stimulation in patients with endometrioma (13, 22). Al-Azemi et al. (23) have reported that ovarian endometrioma led to a decreased response to gonadotropins, as reflected by the smaller number of retrieved oocytes, which was also shown in our study.

Pellicer et al. (24) have demonstrated that endometriosis caused poor quality oocytes and embryos with decreased ability for implantation. Different mechanisms such as changes in autoimmune factors, cytokines or production of growth factors, increased rate of granulosa cell apoptosis, and decreased steroid levels were considered as negative factors for follicular growth and oocyte maturity in these patients (25, 26).

Although some studies have reported poor IVF-embryo transfer (ET) outcomes with endometriosis (9, 11, 12), we could not find any negative impact of ovarian endometrioma on clinical pregnancy and implantation rates. Some scholars have also shown that the existence of endometrioma did not influence embryo implantation, and have proposed that the detrimental effects were limited to the fertilization phase (27).

In patients with unilateral endometrioma, the presence of endometrioma at the time of aspiration did not compromise our ICSI outcomes. This result was in accordance with the study of Almog et al. (28) in which they found a similar number of antral follicles and oocytes retrieved from the affected and healthy contra lateral ovaries. It seems that in women with unilateral disease, the contra lateral intact ovary compensated for ovarian function and fertility potential (14). In the present study, we did not include patients who had surgery for their endometriosis. Additional studies are required to evaluate the effect of surgery.

## Conclusion

Despite the lower response to gonadotropins in endometrioma patients, the rates of pregnancy and implantation rates, embryo quality, total number of embryos transferred per patient and fertilization of MII oocytes were not affected by the presence of the endometrioma. It seems that endometrial receptivity in two groups was similar. However, in consideration of the higher cancelling rate, the prognosis for patients with endometrioma was worse than in patients with male associated infertility.
